# Antibacterial Poly(ε-CL)/Hydroxyapatite Electrospun Fibers Reinforced by Poly(ε-CL)-*b*-poly(ethylene phosphoric acid)

**DOI:** 10.3390/ijms22147690

**Published:** 2021-07-19

**Authors:** Ilya Nifant’ev, Dmitry Gavrilov, Alexander Tavtorkin, Maria Chinova, Victoria Besprozvannykh, Pavel Komarov, Vladimir Zaitsev, Irina Podoprigora, Pavel Ivchenko

**Affiliations:** 1 A.V. Topchiev Institute of Petrochemical Synthesis RAS, Leninsky Pr. 29, 119991 Moscow, Russia; gavrosdm@gmail.com (D.G.); tavtorkin@yandex.ru (A.T.); chinova@yandex.ru (M.C.); vkbesprozvannykh@edu.hse.ru (V.B.); komarrikov@yandex.ru (P.K.); phpasha1@yandex.ru (P.I.); 2 Chemistry Department, M.V. Lomonosov Moscow State University, Leninskie Gory 1–3, 119991 Moscow, Russia; 3 Faculty of Chemistry, National Research University Higher School of Economics, Miasnitskaya Str. 20, 101000 Moscow, Russia; 4 Priorov Central Institute for Trauma and Orthopedics, Priorova Str. 10, 127299 Moscow, Russia; zaitsev-cito@mail.ru; 5 Department of Microbiology and Virology, Institute of Medicine, RUDN University, 8 Miklukho-Maklaya Str. 8, 117198 Moscow, Russia; podoprigora_iv@pfur.ru

**Keywords:** poly(ε-caprolactone), drug release, electrospinning, hydroxyapatite, nanofibers, nanoparticles, polyphosphoesters, ring-opening polymerization, *St. aureus*, vancomycin

## Abstract

In bone surgery and orthopedics, bioresorbable materials can be helpful in bone repair and countering post-op infections. Explicit antibacterial activity, osteoinductive and osteoconductive effects are essential to achieving this objective. Nonwoven electrospun (ES) fibers are receiving the close attention of physicians as promising materials for wound dressing and tissue engineering; potentially, in high contrast with dense materials, ES mats hamper regeneration of the bone extracellular matrix to a lesser extent. The use of the compositions of inherently biodegradable polyesters (poly(ε-caprolactone) PCL, poly(lactoglycolide), etc.), calcium phosphates and antibiotics is highly prospective, but the task of forming ES fibers from such compositions is complicated by the incompatibility of the main organic and inorganic ingredients, polyesters and calcium phosphates. In the present research we report the synthesis of hydroxyapatite (HAp) nanoparticles with uniform morphology, and demonstrate high efficiency of the block copolymer of PCL and poly(ethylene phosphoric acid) (PEPA) as an efficient compatibilizer for PCL/HAp mixtures that are able to form ES fibers with improved mechanical characteristics. The materials obtained in the presence of vancomycin exhibited incremental drug release against *Staphylococcus aureus* (*St. aureus*).

## 1. Introduction

In recent decades, the progress in bioorganic, inorganic, organic and polymer chemistry resulted in the creation of efficient interdisciplinary techniques proposed for bone treatment, including efficient bone substitution and repair after ‘critical-sized’ bone damage caused by trauma, surgery or infection [1,2,3,4,5]. The desired characteristics of the materials for bone substitution and repair are biocompatibility, osteoinductivity and osteoconductivity, sufficient mechanical strength and antibacterial activity. Partially substituted hydroxyapatite (HAp) is a base of the main mineral component of bone [6,7,8], which is why materials based on synthetic HAp and other calcium phosphates with given morphologies are highly prospective [9,10,11,12,13], especially in view of the osteoinductive effect of both Ca^2+^ and PO_4_^3−^ ions [14]. A large number of publications reported good results in the use of synthetic HAp impregnated with antibiotics against pathogens which are the causative agents of osteomyelitis [15,16,17,18,19,20,21]. However, ‘pure HAp’-based materials have a number of shortcomings, in particular, excessive hardness and brittleness [22].

Biodegradable polymers, such as poly(ε-caprolactone) (PCL), poly(lactic acid) (PLA) and poly(lactoglycolide)s (PLGA), are considered prospective components of HAp-based bone substitutes that can potentially provide marked improvement in the mechanical characteristics of composites [23,24]. A number of scientific articles reported notable results regrding the use of dense HAp/polyester composites impregnated with antibiotics [25,26,27,28,29], despite the fact that dense composites can hinder regeneration of the bone extracellular matrix. With that in mind, fibrous composite materials appear promising, however, the low compatibility of calcium phosphates with polyesters is especially visible in the case of attempts to make electrospun (ES) mats from the corresponding composite suspensions [30,31,32,33,34].

**Figure 1:**
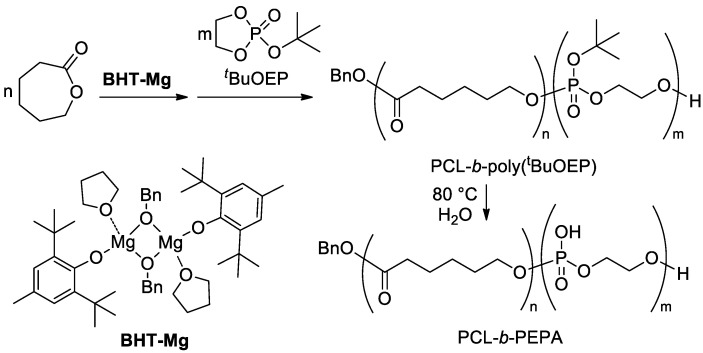
The synthesis of PCL-*b*-PEPA [36].

We propose that the mechanical characteristics of ES materials could be improved by the use of a polymer compatibilizer. Block copolymer, containing poly(ethylene phosphoric acid) (PEPA) fragment (Figure 1), served as a promising choice, especially considering the fact that Ca^2+^ salts of PEPA promote osteogenic differentiation of stem cells [35]. As was shown previously, such copolymers can be obtained by mild thermolysis of PCL-*b*-poly(*^t^*BuOEP) [36], the product of living block copolymerization of εCL and 2-(*tert*-butoxy)-1,3,2-dioxaphospholane 2-oxide (*tert*-butyl ethylene phosphate, *^t^*BuOEP), catalyzed by heteroleptic Mg complex of 2,6-di-*tert*-butyl-4-methylphenol (BHT-H) of formula [(BHT)Mg(μ-OBn)(THF)]_2_ (**BHT-Mg**, Figure 1) [37,38].

In the present work, we report the synthesis and characterization of a nanosized HAp preparation of PCL-*b*-poly(*^t^*BuOEP) with relatively small content of ethylene phosphate subunits, and a comparative study of PCL/HAp composites with and without PCL-*b*-PEPA compatibilizer as a starting material for ES molding. Absorption of vancomycin before and after ES molding was also investigated, and the efficiency of the vancomycin-loaded ES mats against *St. aureus* was evaluated using Kirby–Bauer disk diffusion test.

## 2. Results

### 2.1. Preparation and Characterization of Nanosized HAp

According to the biomimetic principle, synthetic HAp nanoparticles need to be nanosized and have less crystalline structures, similar to those of HAp nanoparticles in natural bones [39]. In the synthesis of HAp, we used the method, proposed by Li et al. [40]. This method is based on the reaction of Ca(NO_3_)_2_ with (NH_4_)_2_HPO_4_ in aqueous media (Equation (1)) at pH 10–12 (the given value of pH is provided by the addition of aq. NH_3_) at different temperatures. The size of HAp nanoparticles depends on the reaction temperature; the length of nanorods increases from 10 to 100 nm with rise of the temperature from 0 to 100 °C [40].

10 Ca^2+^ + 6PO_4_^3−^ + 2OH^−^ → Ca_10_(PO_4_)_6_(OH)_2_ ↓ (1)

**Figure 2:**
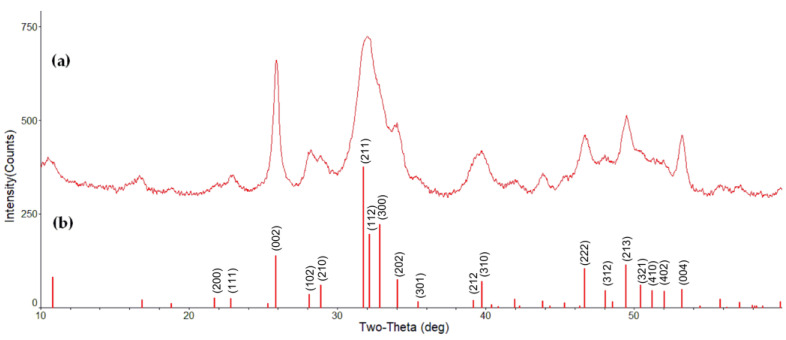
XRD pattern of HAp obtained (**a**) and standard HAp (JCPDS 09-0432) as reference (**b**).

We conducted the reaction at 20 °C for 24 h and obtained a HAp precipitate with 82% yield (for details, see Section 4.1). To check its crystalline structure, the sample was analyzed by X-ray diffraction (XRD). As can be seen from Figure 2, the crystalline peaks of HAp nanoparticles seemed much broader than the sharp peaks of sintered HAp with highly crystallized structures (JCPDS file 09-0432). Nevertheless, XRD pattern of nanosized HAp presented a characteristic diffraction peak motif that is consistent with those of crystalline HAP, and mimics ‘poorly crystalline’ precipitated [40] or bone-derived [41] HAp. All peaks were indexed to a hexagonal lattice of hydroxyapatite crystals.

**Figure 3:**
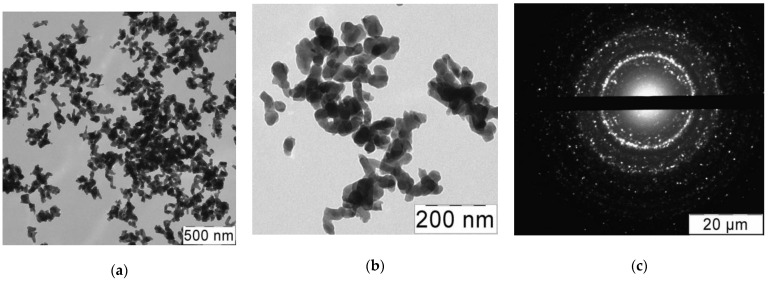
(**a**,**b**) TEM images of HAp nanoparticles; (**c**) electron diffraction pattern of HAp nanoparticles.

From transmission electron microscopy (TEM) data (Figure 3a,b), HAp particles were found to be 50–100 nm long and 20–50 nm wide, which were slightly larger than bone-derived HAp nanoparticles (~40–60 × 20 nm [42]) with close aspect ratio. TEM diffraction pattern (Figure 3c) reflects the substantially amorphous nature of the crystals obtained, in contrast with the well-shaped HAp obtained by the hydrothermal method [43].

### 2.2. Synthesis of PCL-b-poly(^t^BuOEP) 

**Figure 4:**
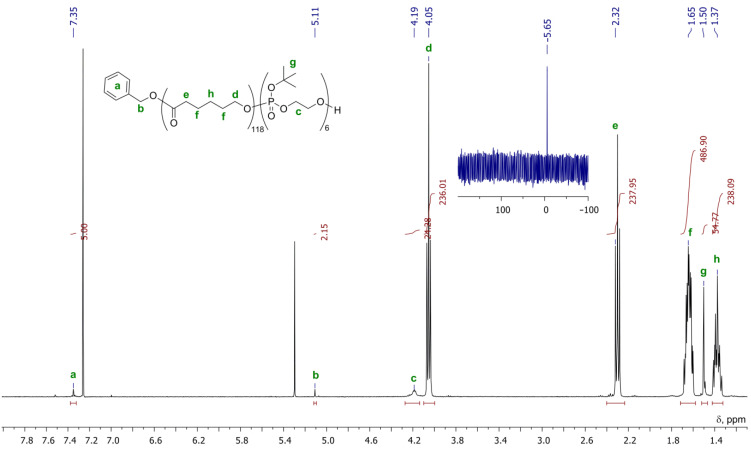
^1^H (black line) and ^31^P (blue line) NMR spectra of BnO-(εCL)_118_-*b*-(*^t^*BuOEP)_6_ recorded in CDCl_3_ at 20 °C.

Block copolymer PCL-*b*-poly(*^t^*BuOEP) was obtained by **BHT-Mg**-initiated fast polymerization of εCL, followed by relatively slow polymerization of *^t^*BuOEP (for experimental details, see Section 4.2). Comonomers were loaded in εCL/*^t^*BuOEP/Mg at a ratio of 100:10:1. As can be seen by end-group analysis of the ^1^H NMR spectrum (Figure 4), separated copolymers matched the formula BnO-(εCL)_118_-*b*-(*^t^*BuOEP)_6_. As was demonstrated previously [36], in protic solvents, such copolymers degrade at relatively low temperatures (~80 °C) with the formation of PEPA copolymers. We propose that transformation of PCL-*b*-poly(*^t^*BuOEP) to PCL-*b*-PEPA can occur during preparation of PCL/HAP composites when using a proton solvent and mildly elevated temperatures (see Section 2.3).

### 2.3. Preparation and ES Molding of PCL/HAp Composites

Electrospinning is a versatile and viable technique for making nanofibrous materials [44,45,46,47,48,49,50]. The formation of ES fibers with given diameters and uniform morphology are determined by processing parameters such as voltage, flow rate and spinneret-collector distance, the chemical nature of the polymer and solvent, as well as conductivity, the dielectric constant and viscoelastic properties of the polymer solutions [45,48].

Encapsulation of calcium phosphates on ES nanofibers has been reviewed very recently by Nathanael and Oh [51]. Preparation of PCL/HAp ES mats with different ratios of its components was reported recently. Aragon et al. used CH_2_Cl_2_/DMF as a solvent; HAp dispersion was stabilized by TWEEN^®^ 180, ES fibers contained 14% of HAp [31]. Stastna et al. used a 3:1 mixture of acetic and formic acids as a solvent without addition of a surfactant [34]; the mechanical characteristics of ES mats were not studied.

**Figure 5:**
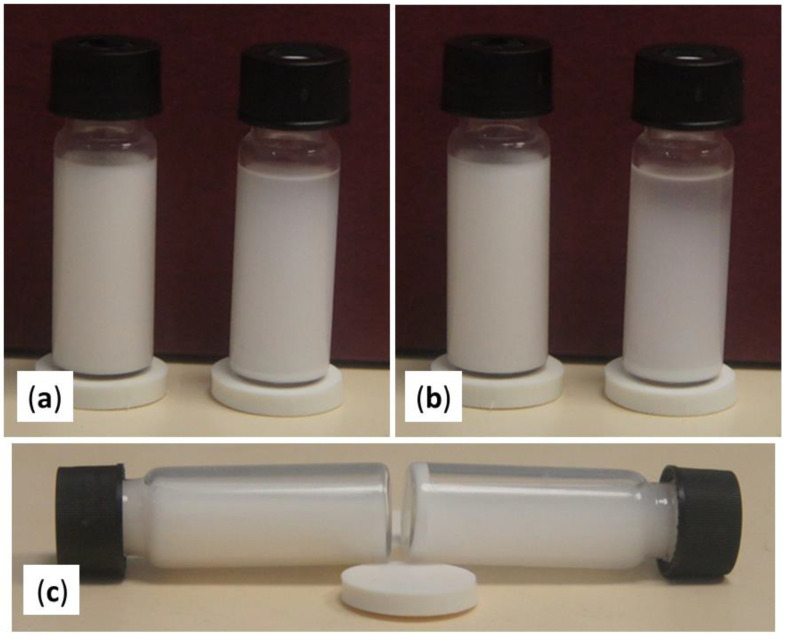
Sedimentation of HAp in the presence (left) and in the absence (right) of PCL-*b*-PEPA: sedimentation after (**a**) 1 h and (**b**) 2 h; (**c**) stable dispersion and precipitate formed after 8 h.

Based on our previous work [52], we used hexafluoroisopropanol (HFIP) as a solvent for PCL and dispersion media for HAp nanoparticles. We proposed that block copolymers of PCL and PEPA could act as an efficient compatibilizers for HAp/PCL composites. We observed that, in the absence of PCL-*b*-PEPA, sedimentation of HAp in HFIP occurred within 2 h, whereas HAp dispersion, prepared in the presence of PCL-*b*-PEPA, was stable for 8 h (Figure 5).

In all ES experiments, we used commercial εCL homopolymer ‘Polymorphus’ (*M*_n_ = 87.5 kDa, *Ð*_M_ = 1.46) as a PCL component, and prepared a series of ES films from pure PCL (Table 1, Entry **C1**), dispersions of HAp in PCL solutions with different PCL/HAp ratios (Table 1, Entries **C2**–**C4**) and dispersions of HAp, prepared by stirring at 90 °C with different amounts of PCL-*b*-poly(*^t^*BuOEP) (this temperature provides transformation to PCL-*b*-PEPA) with subsequent addition of PCL (Table 1, Entries **C5**–**C8**). Experimental details of the preparation and ES molding of PCL/HAp dispersions are presented in Section 4.3.

**Figure 6:**
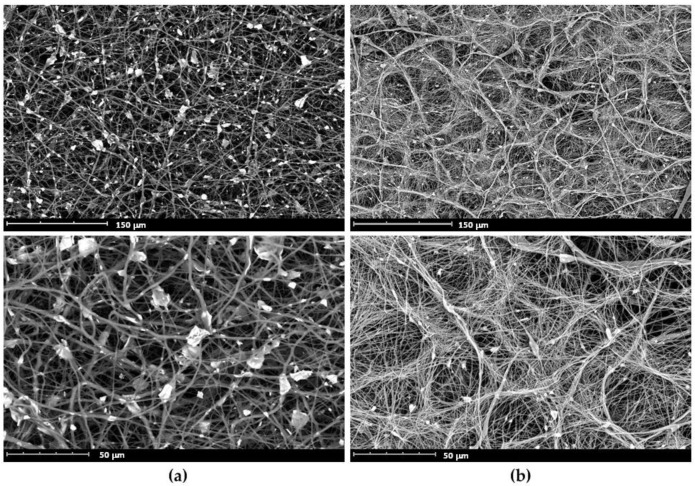
SEM images of PCL/HAp composite ES fibers, prepared (**a**) without PCL-*b*-poly(*^t^*BuOEP) (Table 1, Entry **C3**) and (**b**) in the presence of 5 wt.% PCL-*b*-poly(*^t^*BuOEP) (Table 1, Entry **C6**).

Scanning electron microscopy (SEM) images of the ES mats are illustrated in Figure 6. As can be seen on Figure 6b, the presence of PCL-*b*-PEPA provides efficient dispersing of HAp nanoparticles, whereas in the absence of the additive the aggregation of HAp occurs with a formation of particles of at least 10 μm (Figure 6a).

**Figure 7:**
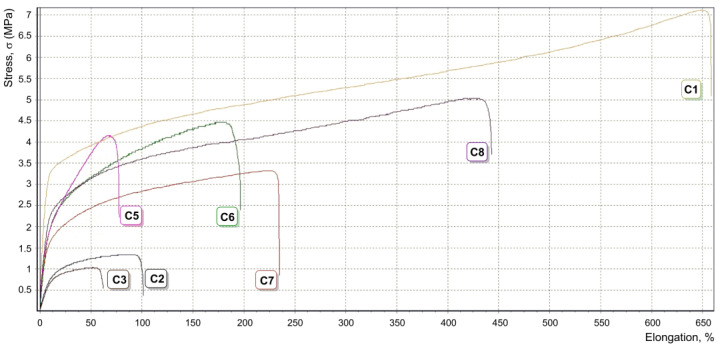
Stress-strain curves of the ES fibers **C1**–**C3**, **C5**–**C8** (Table 1).

The tensile properties of the ES fibrous materials prepared (Table 1) were examined at 25 °C using standard methodology (see Section 4.3). The results of mechanical testing are summarized in Table 1 and illustrated in Figure 7. ES fiber prepared from PCL without addition of HAp (Table 1, Entry **C1**) showed good morphology and mechanical characteristics. Increase of the HAp content from 10% to 15% (Table 1, Entries **C2** and **C3**) resulted in dramatic decreases of both strength and elongation. PCL/HAp composite containing 25 wt.% of HAp (Table 1, Entry **C4**) formed a fragile fibrous film and was therefore not studied. Obviously, the films prepared without compatibilizer are unsuitable for biomedical applications.

The addition of minimal amounts of PCL-*b*-poly(*^t^*BuOEP) to compositions containing 15 wt.% HAp resulted in a substantial rise in strength (Table 1, Entry **C5**), however, the material had low elasticity. Increasing the amount of compatibilizer improved the elasticity (Table 1, Entries **C6** and **C7**). Fibrous material with good characteristics was prepared from suspension containing 5 wt.% HAp (Table 1, Entry **C8**); this film was only marginally behind PCL film in its mechanical characteristics.

### 2.4. ES Fibrous Films Containing Vancomycin

**Figure 8:**
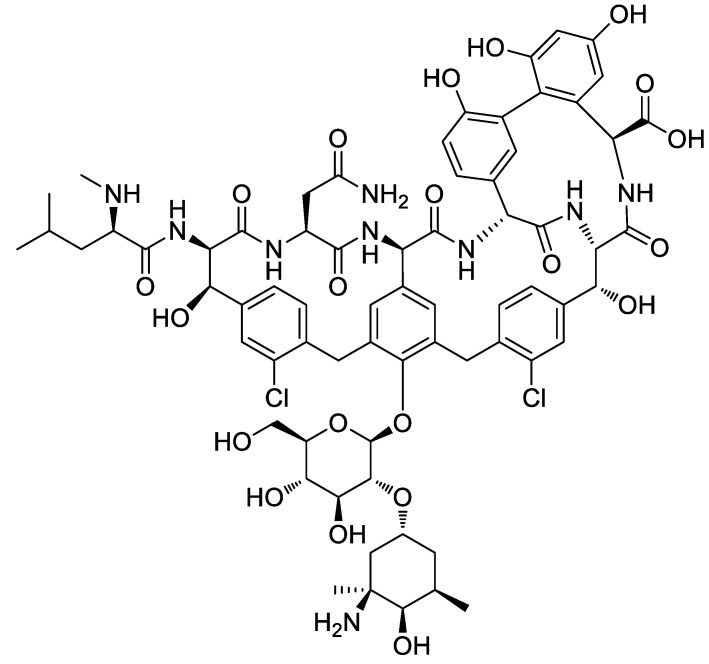
The chemical formula of vancomycin (free base).

Our further studies were aimed at preparation of the ES fibrous films containing vancomycin, and at evaluating of the adsorption and release of this medication. Vancomycin (**Van**) is an antibiotic recommended for the treatment of bone infections, caused by methicillin-resistant *St. aureus* [53] and is classified by the WHO as critically important for human medicine [54]. By its chemical formula (Figure 8), **Van** contains reactive amino, hydroxy and carboxy groups that are potentially able to chemically binding with basic calcium phosphates.

In its neutral form **Van** is not soluble in water, and therefore is used in the form of dihydrochloride, **Van·2HCl**. The first works on the use of **Van**-loaded calcium phosphates were carried out more than 20 years ago [20,21]. Subsequently, **Van**-contained composites of polyesters with calcium phosphates have been studied with marked antibacterial effect [26,55,56,57,58,59].

In our study, we first investigated the ability of the HAp nanoparticles to absorb **Van** in an aqueous medium. The concentration of **Van·2HCl** was determined by UV/vis spectroscopy, and the experiment was conducted using the published method [60]. At the conclusion of a five-day experiment we found that HAp was almost incapable of vancomycin absorption (Table 2).

The high stability of HAp/PCL-*b*-PEPA dispersion in HFIP (Figure 5) raised questions about the cause of colloid stabilization. In general, there are three mechanisms for stabilization of mineral suspensions: electrostatic, steric and electrosteric. Electrostatic stabilization is achieved through the action of repulsive force between colloidal particles that is directly related to the diffuse layer charge on the particles. In steric stabilization, polymer molecules are adsorbed at the surface of particles, thus preventing them from coming into contact with one and other. Electrosteric stabilization is achieved with the involvement of the electrostatic binding of polymer fragments with the surface of the colloidal particles [61,62].

Electrostatic stabilization can be detected by experimental determination of zeta potential (*ξ*), or the electrical potential of the shear plane at the surfaces of colloidal particles. The value and the sign of the charge of *ξ* can be derived from measurements of the electrophoretic mobilities of the suspended particles. We prepared three samples of HAp dispersions in HFIP with 500 ppm concentrations: (1) without additives; with an addition of 40 wt.% of PCL-*b*-poly(*^t^*BuOEP); and with an addition of 40 wt.% of PCL-*b*-poly(*^t^*BuOEP) and 20 wt.% of **Van·2HCl**. The two latter samples were subjected to 10 min of heating at 90 °C to form PCL-*b*-PEPA.

The results of *ξ*-potential measurements at 20 °C (see Section 4.4.2) showed decrease in the *ξ* value in a row, +36.7 mV (1), +11.6 mV (2) and +4.2 mV (3), confirming the chemical binding of PCL-*b*-PEPA with the HAp surface. Note that the value of *ξ*-potential of HAp in HFIP was found to be close to the value of *ξ*-potential of HAp in isopropanol, +38.2 mV [63]. The values of electrophoretic mobility were 0.29, 0.09 and 0.03 µmcm/Vs for (1), (2) and (3), respectively. The values of conductivity were 1.3, 12.4 and 0.5 μS/cm for (1), (2) and (3), respectively. The very low value of conductivity for the dispersion of **Van·2HCl** loaded in HAp/PCL-*b*-PEPA suspension confirms the efficient binding of the drug with HAp particles.

With the aim of studying vancomycin’s release from ES films, we prepared four samples of fibrous mats (Table 3). Sample **V1** was ES fibrous film, molded from the solution, containing PCL and **Van·2HCl** in HFIP. The samples **V2** and **V3** (Table 3) were prepared by ES molding of HAp dispersions in PCL/PCL*-b*-PEPA/**Van** solutions. Finally, sample **V4** (Table 3) was obtained by the exposition of the previously obtained ES film (Table 1, Entry **C6**) in aqueous solution of **Van·2HCl**. The details of the experiments are presented in Section 4.4.2.

**Figure 9:**
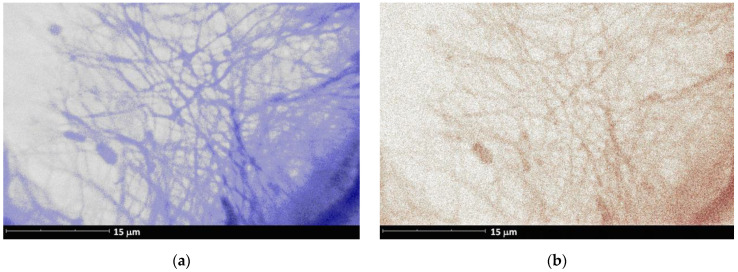
The distribution of (**a**) Ca and (**b**) N in ES fibrous film **V3** (SEM EDX mapping data).

Figure 9’s example of **V3** illustrates a distribution of Ca in ES fibrous mats that is fully consistent with the distribution of N (EDX data for the corresponding SEM study). Taking into account surface modification of HAp by PCL-*b*-PEPA and the fact that **Van** is the only source of nitrogen in the reaction mixture, this distribution confirms the binding of **Van** and PCL-*b*-PEPA-treated HAp.

Kirby–Bauer disk diffusion tests were performed to determine the time dependence of the antimicrobial activity of **V1**–**V4**. In our studies we used a clinical strain of *St. aureus*; experimental details are presented in Section 4.4.

**Figure 10:**
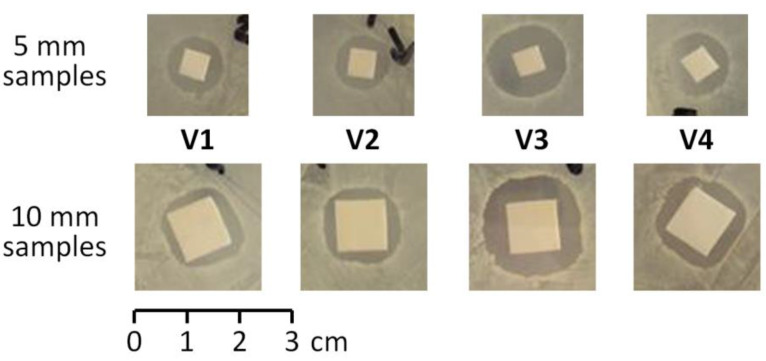
Inhibition of *St. aureus* by **V1**–**V4** film samples during Kirby–Bauer disk diffusion tests (24 h incubation at 37 °C). The sample V3 demonstrated maximum effect.

In the first series of the experiments, we estimated the efficiency of 5 × 5 mm and 10 × 10 mm film samples against *St. aureus* seeded on Mueller–Hinton agar plates during Kirby–Bauer disk diffusion test after 24 h of incubation at 37 °C. The zone of inhibition was determined by measuring the diameter of the area around the sample that remained free from microbial growth (Figure 10); the test was performed in triplicate for each type of material (Table 4).

In the second series of the experiments, the procedure was performed for the samples that had partially released vancomycin load to determine whether there was sufficient residual **Van** to inhibit bacterial growth. To this end, **Van**-containing 5 × 5 mm film samples were immersed in saline (100 μL), with daily replacement of the medium for 1, 2 and 3 days. The media (1-, 2- and 3-day extracts) and the films (after 3 days) were studied by diffusion test (Table 5). These experiments showed that high efficiency in drug release had been demonstrated by PCL/HAp fibrous mats prepared from **Van**-containing spinneret mixtures.

## 3. Discussion

The results of our experiments on ES molding of PCL/HAp composites demonstrate that the addition of PCL-*b*-PEPA compatibilizer considerably improved the mechanical characteristics of the materials obtained. It may be assumed that PEPA fragments serve as dispersants for HAp, thus preventing agglomeration of the HAp nanoparticles (Figure 5b), that was observed in the absence of the PEPA-containing additive (Figure 5a). Apparently, the stabilization of HAp dispersion by PCL-*b*-PEPA cannot be explained by electrostatic factors (as evidenced by *ξ*-potential measurements). We assume that PCL-*b*-PEPA stabilize HAp dispersion by an electrosteric mechanism, which implies chemical binding between the HAp surface and the phosphate block of PCL-*b*-PEPA. Note that such binding was demonstrated previously for HAp and low-*M*_W_ organophosphates and organophosphonates [64,65].

In our experiments, HAp nanoparticles demonstrated relatively low absorption capacity for **Van·2HCl** from aqueous medium. However, in the presence of PCL-*b*-PEPA we observed efficient binding of **Van·2HCl** by HAP. The addition of **Van** at the stage of the preparation of spinneret composition in the presence of PCL-*b*-PEPA provided the formation of the fibrous materials that contain antibiotic; the distributions of Ca and N in EDX map are nearly identical.

Additionally, we observed relatively slow release of the **Van** during in vitro experiments with HAp-loaded ES films **V2** and **V3**. We propose that this result can be explained by a chemical binding of PCL-*b*-PEPA with HAp that may increase the absorption capacity of HAp wih respect to **Van** due to additional binding between he amino group of **Van** with the phosphate group in PEPA blocks and lipophilic fragments of **Van** with PCL blocks. In addition, ES fibers, obtained in the presence of PCL-*b*-PEPA, contain HAp particles that are substantially layered by PCL (Figure 6b), slowing down the release of the drug, located near the HAp surface. It should also be noted that ready-made HAp/PCL-*b*-PEPA/PCL ES fibrous material **V4** had not shown potential for binding and release of **Van**.

## 4. Materials and Methods

### 4.1. Preparation and Characterization of HAp Nanoparticles

#### 4.1.1. Reagents and Equipment

Ca(NO_3_)_2_·4H_2_O, 85% aq. H_3_PO_4_, 25% aq. NH_3_ and ethanol (Prime Chemicals Group, Moscow, Russia) were used as purchased.

XRD patterns were obtained with a Rotaflex RU-200 X-ray source (Rigaku, Japan) with rotating anode tube (CuKα radiation, 50 kV and 160 mA mode) equipped with a horizontal wide-angle goniometer Rigaku D/Max-RC with Bragg-Brentano θ–2θ geometry at angle range 2θ = 10–60°, step 0.02°, continuous scan rate 1°/min.

Sample morphologies of HAp nanoparticles were studied using a Hitachi HT7700 transmission electron microscope (Hitachi Ltd., Tokyo, Japan). Images were acquired in bright-field TEM mode at 100 kV accelerating voltage. A target-oriented approach was utilized for the optimization of the analytic measurements [66]. Before measurement, the samples were mounted on a 3 mm copper grid and fixed in a grid holder.

#### 4.1.2. Synthesis of HAp Nanoparticles

Nanosized HAp was prepared via a chemical precipitation approach [40]. Aqueous solutions of Ca(NO_3_)_2_·4H_2_O (100 mL, 0.2 M) and H_3_PO_4_ (60 mL, 0.2 M) were prepared separately. The aqueous solution of Ca(NO_3_)_2_ was then poured into the phosphate solution with stirring, followed by the addition of the NH_3_ solution to adjust the pH value to 11. The reaction was maintained at 20 °C for 24 h, the precipitate was washed with water (5 × 100 mL), then with ethanol (3 × 50 mL) and dried *in vacuo*. The yield was 1.65 g (82%).

### 4.2. Synthesis of PCL-b-poly(^t^BuOEP)

#### 4.2.1. Reagents and Equipment

Tetrahydrofuran (THF), diethyl ether and triethylamine (Merck, Kenilworth, NJ, USA) were refluxed with Na/benzophenone and distilled prior to use. Benzene (Merck, Kenilworth, NJ, USA) was distilled with Na/benzophenone/dibenzo-18-crown-6. Dichloromethane was refluxed over CaH_2_ and distilled under argon atmosphere. 2,6-Di-*tert*-butyl-4-methylphenol (BHT-H, ≥99), di-*n*-butylmagnesium (1.0 M solution in heptane), PCl_3_ (≥99.9%), ethylene glycol (≥99.9%) and acetic acid (≥99.9%, Merck, Kenilworth, NJ, USA) were used as purchased. *tert*-Butyl alcohol (99%, Merck, Kenilworth, NJ, USA) was distilled over BaO and stored under argon. Also, 2-Chloro-2-oxo-1,3,2-dioxaphospholane [67], *^t^*BuOEP [38] and **BHT-Mg** [37] were prepared according to previously described methods.

CDCl_3_ (D 99.8%, Cambridge Isotope Laboratories, Inc., Cambridge, MS, USA) was distilled over P_2_O_5_ and stored over 4 Å molecular sieves. The ^1^H (400 MHz) and ^31^P (162 MHz) NMR spectra were recorded on a Bruker AVANCE 400 spectrometer (Bruker, Billerica, MS, USA) at 20 °C. Chemical shifts were reported relative to the solvent residual peak (*δ* = 7.26 ppm).

Size exclusion chromatography (SEC) was performed on a 1260 Infinity II chromatograph (Agilent Technologies, CA, USA) equipped with a PLgel MIXED C column, using THF as an eluent (1 mL/min). The measurements were recorded with universal calibration according to polystyrene standards at 30 °C.

#### 4.2.2. Synthesis of PCL-b-poly(*^t^*BuOEP)

The polymerization experiment was carried out under an argon atmosphere. **BHT-Mg** (10.6 mg, 0.25 mmol Mg) was added to the solution of εCL (2.85 g, 25 mmol) in CH_2_Cl_2_ (10 mL). After 3 h of stirring at 20 °C, *^t^*BuOEP (0.45 g, 2.5 mmol) was added. After 30 h AcOH (20 μL) was added, the mixture was poured into pentane (10 mL). The polymer was separated by centrifugation and decantation, dissolved in CH_2_Cl_2_ (1 mL), reprecipitated again, separated and dried at 0.01 Torr to constant weight. The yield was 2.73 g (82%), white solid, *M_n_* (SEC) = 13.8·10^3^ Da, *M*_n_ (NMR) = 14.7·10^3^ Da, *Ð_M_* = 1.42. ^1^H NMR (400 MHz, CDCl_3_, 20 °C): δ 7.35 (m, 5H, Ph); 5.11 (s, 2H, PhCH_2_); 4.19 (m, 24H, POCH_2_); 4.05 (t, ^3^*J* = 6.8 Hz, 236H); 2.32 (t, ^3^*J* = 7.4 Hz, 238H); 1.65 (m, 486H); 1.50 (s, 55H, *^t^*Bu); 1.37 (m, 238H). ^31^P {^1^H} NMR (162 MHz, CDCl_3_, 20 °C): δ −5.65.

### 4.3. Preparation and Study of ES Mats

#### 4.3.1. Reagents and Equipment

PCL (Polymorph, Bright China Industry Inc., Shenzhen, China) and HFIP (99.5%, P&M Invest, Moscow, Russia) were used as purchased.

ES was performed with a given flow rate using a 10 mL syringe with spinneret (0.8 mm diameter needle) and collector (12 × 12 cm square aluminum foil). The distance between spinneret and collector was 10 cm and the potential difference was 10 kV. The motion of the collector was provided by a stepping motor (speed 5 mm/s, amplitude 4 cm).

SEM images were obtained using a Phenom XL G2 equipped with EDX detection (Thermo Fisher Scientific, Waltham, MS, USA) at the accelerating voltage of 15.0 kV.

Universal tensile testing machine I1140M-5-01-1 (Tochpribor-KB, Ivanovo, Russia) and ASTM D638 method were used for ES film mechanical testing. Dog-bone tensile specimens were prepared by punching the electrospun fiber films using a stainless steel die (ASTM standard D1708-96, 22 × 5 mm).

#### 4.3.2. Preparation of ES Mats

The sample **C1** was prepared by dissolution of PCL (1 g) in HFIP (4 mL); the ES flow rate was 0.70 mL/h.

The sample **C2**. PCL (900 mg) was dissolved in HFIP (4 mL); HAp (100 mg) was added with stirring. After 30 min the suspension was transferred into the spinneret and the ES flow rate was 0.36 mL/h. The samples **C3** and **C4** were prepared by the same manner, with the loading of 850 mg PCL/150 mg HAp (**C3**) and 750 mg PCL/250 mg HAp (**C4**).

For sample **C5** PCL-*b*-poly(*^t^*BuOEP) (25 mg) was dissolved in HFIP (4 mL) and HAp (150 mg) was added with stirring. The mixture was heated for 15 min at 90 °C. After cooling to the room temperature, PCL (825 mg) was added. After 15 min the suspension was transferred into the spinneret; the ES flow rate was 0.72 mL/h. Samples **C6**–**C8** were prepared by the same manner with the loading of 800 mg PCL/150 mg HAp/50 mg PCL-*b*-poly(*^t^*BuOEP) (**C6**), 775 mg PCL/150 mg HAp/75 mg PCL-*b*-poly(*^t^*BuOEP) (**C7**), and 925 mg PCL/50 mg HAp/25 mg PCL-*b*-poly(*^t^*BuOEP) (**C8**).

### 4.4. Experiments with Van-Loaded HAp Particles and ES Films

#### 4.4.1. Reagents and Equipment

**Van·2HCl** (Elfa Laboratories, Haryana, India) was used as purchased. A *St. aureus* strain, deposited in the collection of microorganism cultures of the chair of microbiology and virology of RUDN University, was used in this study. *St. aureus* was grown in Brain Heart Infusion broth (HiMedia Laboratories Pvt. Ltd., Mumbai, India), at 37 °C and overnight, on shaker conditions set at 200 rpm. The *St. aureus* culture was then washed by centrifugation and the sediment was resuspended with 0.9% sodium chloride solution. The concentration of the microbial suspension was adjusted using the McFarland 0.5 turbidity standard. Finally, 100 µL of a standardized suspension was seeded by the “lawn” method using a 60 mm and 90 mm petri dish with a Mueller–Hinton medium (HiMedia Laboratories Pvt. Ltd., Mumbai, India).

#### 4.4.2. ζ-Potential Measurements

The electrokinetic potential (*ξ*-potential), electroconductivity and electrophoretic mobility of HAp and modified HAp particle dispersions were estimated by dynamic light scattering (DLS) using the two-angle particle and molecule size analyzer Zetasizer Nano ZS (Malvern Panalytical, Malvern, UK) equipped with a DTS1060 particle diameter cell. The measurements were carried out for the suspensions containing 0.5 mg of solid per 1 mL of the HFIP. The software of the instrument provides the ζ-potential from electrophoretic mobilities (μE), using the Henry Equation (2).

(2) $$U_{E}=\frac{2\varepsilon \xi f(ka)}{3\eta }$$

where *U_E_*—electrophoretic mobility, *ε*—dielectric constant of the medium (15.7 for HFIP), *ξ*—zeta potential, *η*—viscosity (1.65 cP at 20 °C for HFIP), *f*(*ka*)—Henry function.

#### 4.4.3. Preparation of Vancomycin-Loaded ES Films

The sample **V1** was prepared by dissolution of PCL (975 mg) and **Van·2HCl** (25 mg) in HFIP (4 mL), the ES flow rate was 1.07 mL/h.

The sample **V2**. PCL-*b*-poly(*^t^*BuOEP) (50 mg), HAp (150 mg) and HFIP (2 mL) were stirred for 15 min at 90 °C. After cooling to room temperature, the solution of **Van** (free base, 25 mg) and PCL (775 mg) in HFIP (2 mL) was added. After 15 min of stirring the suspension was transferred into the spinneret, the ES flow rate was 0.71 mL/h.

The sample **V3**. PCL-*b*-poly(*^t^*BuOEP) (50 mg) was dissolved in HFIP (4 mL). The solution was stirred for 15 min at 90 °C to obtain PCL-*b*-PEPA solution. Separately, HAp (150 mg) and **Van·2HCl** (25 mg) were stirred for 30 min in water (1 mL), evaporated under reduced pressure and dried *in vacuo*. This precipitate and PCL (775 mg) were added to the solution of PCL-*b*-PEPA. After 15 min of stirring the suspension was transferred into the spinneret, the ES flow rate was 1.07 mL/h.

The sample **V4** was obtained by the 12 h exposition of the previously obtained ES film (Table 1, Entry 6) in the solution of 1.0 g of **Van·2HCl** in water (100 mL), followed by 30 min exposition in 100 mL of water and drying.

#### 4.4.4. Antibacterial Activity of ES Films

In the first series of the experiments, 5 × 5 mm and 10 × 10 mm samples of the fibrous films **V1**–**V4** were placed on top of the agar with sterile forceps. The plates were incubated at 37 °C and 5% CO_2_ for 24 h and zones of inhibition (ZOI) were subsequently analyzed and recorded.

In the second series of the experiments, 5 × 5 mm were placed into vials (1.5 mL) containing 100 μL saline and stored for 24 h at 37 °C. The solution was eliminated, and the procedure was repeated twice with the obtaining of 2- and 3-day extracts. The antibacterial activities of the extracts and residual samples of ES films were estimated using Kirby–Bauer test.

## 5. Conclusions

In our study, we demonstrated a marked efficiency of the use of PEPA-containing poly(εCL) compatibilizer for improving the characteristics of PCL/HAp composites. It is entirely possible that a PCL/HAp combination is not the best choice among polyester/calcium phosphates; many other polyesters (for example, PLGA etc.) have better mechanical characteristics and higher biodegradability and many other calcium phosphates (for example, tricalcium phosphate, carbonated apatite etc.) outperform HAp by bioresorbability and/or sorption capacity. Considering that block copolymers of PEPA can be obtained for different types of polyesters, the synthetic approach presented in this work can be successfully applied for the further development of prospective materials for bone surgery and orthopedics.

## Figures and Tables

**Table 1:** Preparation and properties of ES mats (4 mL HFIP was used in all experiments).

Entry	PCL, mg	HAp, mg	PCL-*b*-poly (*^t^*BuOEP), mg	Average Cross-Sectional Area, mm^2^	Tensile Strength, MPa	Yield Stress σ, MPa	Young’s Modulus E, MPa	Elongation at Break εp, %
**C1**	1000	0	0	0.66	4.5 ± 1.0	6.1 ± 1.1	24.1 ± 3.4	676 ± 28
**C2**	900	100	0	0.50	0.44 ± 0.09	0.69 ± 0.09	<7.0	98.6 ± 4.1
**C3**	850	150	0	0.65	0.54 ± 0.02	0.63 ± 0.02	<7.0	65.4 ± 9.5
**C4**	750	250	0	0.68	n.d. ^1^	n.d.	n.d.	n.d.
**C5**	825	150	25	0.13	4.34 ± 0.69	3.30 ± 0.85	34.5 ± 6.2	79 ± 10
**C6**	800	150	50	0.30	2.75 ± 0.63	1.63 ± 0.16	18.0 ± 2.2	213 ± 52
**C7**	775	150	75	0.68	1.91 ± 0.54	1.48 ± 0.30	21.1 ± 5.9	287 ± 19
**C8**	925	50	25	0.44	3.61 ± 0.95	3.1 ± 2.0	19.0 ± 5.9	437 ± 89

^1^ Not determined, fragile film.

**Table 2:** Absorption of **Van·2HCl** (100 mg) by HAp (100 mg) in 5 mL of water.

Time, h	[Van·2HCl], mg/mL
0	22.5
1	22.0
2	21.6
3	21.5
5	19.6
24	20.9
48	19.7
120	19.2

**Table 3:** Vancomycin-loaded ES fibrous films.

Sample	ES Fiber Components, wt.%				Note
	Van or Van·2HCl	PCL	PCL-*b*-poly (*^t^*BuOEP)	HAp	
**V1**	2.5	97.5	0	0	Dissolution of both components; ES
**V2**	2.5	77.5	5.0	15.0	Addition of **Van** + PCL solution to HAp + PCL-*b*-PEPA suspension; ES
**V3**	2.5	77.5	5.0	15.0	Addition of **Van·2HCl** + HAp (dried) and PCL solution to PCL-*b*-PEPA; ES
**V4**	0 ^1^	80.0	5.0	15.0	Exposition of ES film in aq. **Van·2HCl**

^1^ Prepared by 12 h exposition of ES film in 1% aqueous solution of Van·2HCl.

**Table 4:** Inhibition of *St. aureus* by **V1**–**V4** film samples (by diameter of transparent area, mm).

Exp.	V1	V2	V3	V4
Inhibition zone diameter (5 × 5 mm sample), 60 mm Petri dishes				
1	10	11	15	13
2	11	10	16	13
3	11	11	15	12
Average	10.6	10.6	15.3	12.6
Inhibition zone diameter (10 × 10 mm sample), 90 mm Petri dishes				
1	15	15	21	17
2	16	14	21	19
3	15	15	20	16
Average	15.3	14.6	20.7	17.3

**Table 5:** Inhibition of *St. aureus* by **V1**–**V4** film extracts (by diameter of transparent area, mm).

Exp.	1 Day Extract ^1^	2 Day Extract	3 Day Extract	3 Day Film
**V1**	6	0	0	0
**V2**	9	6	5	7
**V3**	12	7	5	5
**V4**	9	0	0	0

^1^ Extracts were studied by the addition of 5 μL of the solution into 2 mm diameter holes in Mueller–Hinton agar.
